# Optimization of Vibration Pretreatment Microwave Curing in Composite Laminate Molding Process

**DOI:** 10.3390/polym15020296

**Published:** 2023-01-06

**Authors:** Dechao Zhang, Lihua Zhan, Chenglong Guan, Jinzhan Guo, Bolin Ma, Guangming Dai, Shunming Yao

**Affiliations:** 1 Light Alloys Research Institute, Central South University, Changsha 410083, China; 2 College of Mechanical and Electrical Engineering, Central South University, Changsha 410083, China

**Keywords:** out-of-autoclave, microwave curing, vibration pretreatment, voids, orthogonal experiment, components, mechanical property

## Abstract

Vibration pretreatment microwave curing is a high-quality and efficient composite out-of-autoclave molding process. Focusing on interlaminar shear strength, the effects of pretreatment temperature, pretreatment time and vibration acceleration on the molding performance of composite components were analyzed sequentially using the orthogonal test design method; a scanning electron microscope (SEM) and optical digital microscope (ODM) were used to analyze the void content and fiber-resin bonding state of the specimens under different curing and molding processes. The results show that the influence order of the different vibration process parameters on the molding quality of the components was: vibration acceleration > pretreatment temperature > pretreatment time. Within the parameters analyzed in this study, the optimal vibration pretreatment process parameters were: pretreatment temperature of 90 °C, pretreatment time of 30 min, and vibration acceleration of 10 g. Using these parameters, the interlaminar shear strength of the component was 82.12 MPa and the void content was 0.37%. Compared with the microwave curing process, the void content decreased by 71.8%, and the interlaminar shear strength increased by 31.6%. The microscopic morphology and mechanical properties basically reached the same level as the standard autoclave process, which achieved a high-quality out-of-autoclave curing and molding manufacturing of aerospace composite components.

## 1. Introduction

Because of their high specific strength and high specific stiffness, carbon-fiber-reinforced resin matrix composites are widely used in the aerospace field [1,2,3]. In addition, some polymer materials can also be used in biomedical applications because of their high strength and harmlessness, such as in dental, obstetric and gynecological surgery as well as in cardiology and orthopedics [4,5,6,7]. Currently, the common curing method for high-performance aerospace composite components is the traditional autoclave curing process [3,8]. However, the traditional autoclave curing process for this material is limited by component forming size and results in high energy consumption and a long curing cycle. Therefore, the new out-of-autoclave curing methods, such as resin-transfer molding (RTM) [9,10], microwave curing [11,12,13], laser curing [14,15], electron beam curing [16,17,18,19] and ultraviolet curing [20,21,22,23], etc., have been explored by many researchers.

With the resin-transfer molding (RTM) process, a series of carbon/phenol composites were prepared by Lee et al. at different injection and molding temperatures, and the mechanical properties of the composites were evaluated [9]. Babu et al. [10] prepared kenaf fiber polyester composite laminates using compression molding technology, vacuum-assisted resin infusion technology and resin-transfer molding technology and evaluated the void content and water absorption of the laminates under the three curing processes. The results show that the specimen under the compression molding process had the highest void content and water absorption.

The research aspects of microwave curing are as follows. Joshi and Bhudolia [11] conducted microwave curing on L-930 carbon-fiber-reinforced polymer prepreg, and the test data showed that the microwave curing process can save over 75% more energy than the autoclave curing process, but the tensile and bending properties of the specimen were reduced by 2–10%. The research presented by Kong et al. [12] compared the microwave curing process and thermal curing process based on a SIO_2_/UPR composite system and demonstrated that microwave curing has characteristics of less time consumption. Meanwhile, a similar result was demonstrated by Rao et al. [13], who prepared glass–epoxy composite laminates by applying the microwave curing process and conventional thermal curing process.

Researchers have conducted a small amount of research on laser curing. Tu et al. [14] proposed a method for the laser-induced curing of composites dependent on transfer printing, and the results of the study showed that the mechanical properties and curing degree of LIG-cured FRC were comparable to those of conventional oven curing with an energy reduction of 10.61%. Wang et al. [15] used infrared laser radiation and a tunnel kiln oven to heat glass-fiber composite rods, and the results of mechanical property testing and microscopic analysis showed that the tensile strength of the laser-radiation-cured rods was about one-fourth higher than that of the kiln-oven-cured rods, and they attributed the difference in mechanical properties to more resin being attached to the fiber surface in the laser-radiation-cured GFRP rods.

An electron beam was used by Bao et al. [16] to cure composite components, and the formed specimen was able to replace the polyimide composites used in the original aero engines without loss of mechanical performance. Abliz et al. [17] established a low-energy electron beam and automatic tape-release curing system in order to achieve a method for the homogeneous out-of-autoclave curing of composites; meanwhile, the effects of exposure dose and post-curing on the interlaminar shear strength (ILSS) of the composites were investigated. The results showed that the maximum interlaminar shear strength of the specimens was 64.7 MPa at an electron beam dose of 50 kGy and a curing temperature of 180 °C for 30 min. MI et al. [18] systematically investigated the difference in the electron beam absorption of cap composite materials due to bending degree using a combination of theory and experiment. The results show that reducing the thickness of the composites at the same degree of bending can improve the uniformity of electron-beam energy absorption. Rizzolo et al. [19] propose a new composite curing process combining vacuum infusion and an electron beam. The results of the study show that the process can be used for the experimental low-cycle manufacturing of aerospace parts by measuring the fiber and pore volume fractions of the specimens.

The ultraviolet (UV) curing method was applied by Compston [20] to form glass fiber/vinyl ester composite components. With less time consumed, the specimens’ properties under the ultraviolet (UV) curing process were similar to those under the room-temperature curing process. Park et al. [21] successfully prepared polymeric nanocomposite molds with high strength and durability using a UV curing process, and the study showed that polymeric nanocomposites promise to be an extremely useful material for fabricating nanopatterned molds using the UV process. Zhang et al. [22] studied the effect of exposure dose on the curing uniformity and interlaminar shear strength of composite materials under UV curing, and the results showed that curing uniformity improves with an increasing exposure dose in a certain range, but interlaminar shear strength decreases with an excessive exposure dose. Jang et al. [23] cured four types of composites using catalysts and UV light. The results demonstrated that UV curing not only saved 70% of the curing time but also had better mechanical properties.

It can be found from the above-mentioned out-of-autoclave curing processes that less time is consumed than in the traditional autoclave curing process. However, because of the lower pressure (<0.1 MPa) in the out-of-autoclave curing process, the void content is uncontrollable; consequently, the specimen quality is influenced. In order to decrease the void content during the out-of-autoclave curing process, the researcher conducted many tests. A low-frequency simple harmonic vibration was introduced by Muric-Nesic et al. [24] into the UV curing process of composites, and a specimen with a void content less than 0.3% was formed using UV curing when vibration pretreatment was performed at room temperature. Meier et al. [25] introduced a low-frequency vibration of 10Hz into the vacuum resin infusion process; the internal void content was reduced to 0.69% and the interlaminar shear strength increased by 10%. Furthermore, the random vibration pretreatment was applied by Yang et al. [26,27] to cure composite components for aerospace applications. The results show that, compared with the vibration-free process, the components’ void content reduces from 6.66% to 0.44%, and the interlaminar shear strength increases from 50.06 MPa to 97.12 MPa. As the above-mentioned works have shown, introducing vibration into the out-of-autoclave curing process of composite materials is an effective means of reducing the void content and improving the mechanical properties.

Following the above study, Guan et al. [28,29] innovatively proposed the introduction of vibration pretreatment into the microwave curing process of composite materials, on the one hand, to cope with the traditional hot air circulation heating method resulting in low heating efficiency and high energy consumption, etc. The use of microwave selective heating characteristics to achieve the rapid and uniform heating of carbon-fiber-reinforced resin matrix composites “within the volume” resulted in a significant reduction in energy consumption and curing time. On the other hand, to cope with the lack of curing pressure in the environment of the composite out-of-autoclave molding process, pores and delamination defects can not be eliminated, and a vibration energy field can be used to reduce the dependence of the composite’s molding quality on the curing pressure.

However, the above research has not further analyzed the influence of important parameters of the composite molding process, such as pretreatment temperature, pretreatment time and vibration acceleration on the molding quality of the components, and it has not explored the applicability of the process to different composite systems. Therefore, to achieve low-pressure, high-quality curing, this study intends to use the orthogonal test design method to investigate the influence of different vibration pretreatment process parameters (pretreatment temperature, pretreatment time, vibration acceleration) on the molding performance of T800/#602 composite materials based on the evaluation of interlayer shear strength combined with extreme difference analysis and ANOVA. SEM and ODM were used to characterize the microscopic morphology of the components made by different curing processes, and the differences in interlaminar shear strength were analyzed in terms of void content and fiber-resin bonding properties. The results of this paper provide a theoretical basis for efficient and high-quality control of the subsequent composite curing process, enriching and developing the theory of out-of-autoclave curing.

## 2. Materials and Methods

The material of this study was carbon-fiber-reinforced resin-based aerospace prepreg provided by Aerospace Changzheng Rui Te Technology Co., Ltd., (Tianjin, China) with the grade T800/#602, a fiber volume fraction of 60%, #602 thermosetting epoxy resin, a prepreg bulk density of 1.6 g/cm^3^, and a single layer thickness of 0.17 mm. This study mainly focuses on composite laminates as the research object, using hand lay-up, with a prepreg size of 200 mm × 200 mm; the number of layers was 14, the lay-up angle parameter was [0/90/0/90/0/90/0]s, and the temperature measurement fiber was embedded in the composite material during the lay-up process for subsequent microwave curing and temperature monitoring.

### 2.1. Microwave and Vibration Equipment

**Figure 1:**
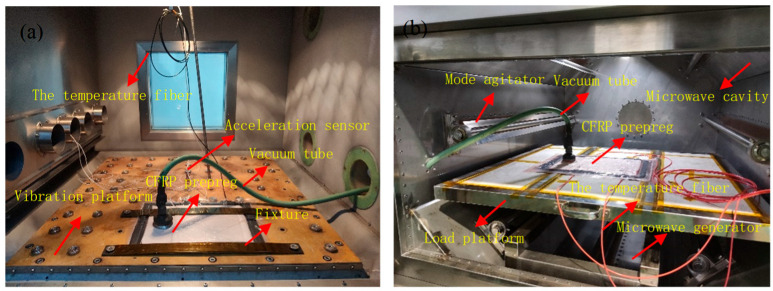
Experimental platform for vibration and microwave curing of composite, (**a**) laminate in vibration processing platform, (**b**) laminate in microwave heating chamber.

The vibration platform and the microwave curing equipment used in this study are shown in Figure 1. The composite laminates were pretreated on the vibration platform and then cured on the microwave platform, and the two experimental platform belong to Central South University in Changsha, Hunan, China. During the entire curing process, vacuum bagging pressure was always present.

### 2.2. Curing Process

Besides the vibration pretreatment microwave curing, autoclave curing (the standard curing process for composite components) and microwave curing were also conducted to clear the influence of the vibration pretreatment process on curing quality.

**Figure 2:**
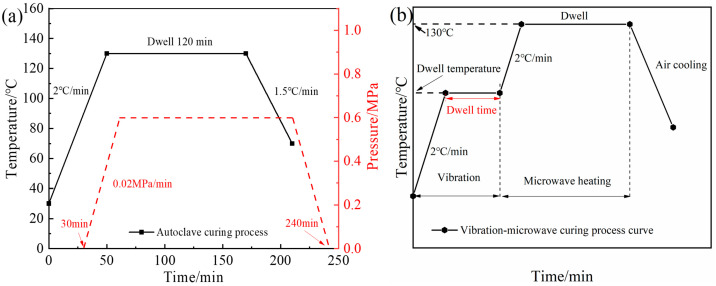
Composite curing process curve, (**a**) autoclave curing process, (**b**) vibration pretreatment microwave curing process.

For the autoclave curing process curve (shown in Figure 2a), the composite laminates were heated from room temperature to 130 °C at the rate of 2 °C/min and then kept for 120 min. The pressure began to increase to 0.6 MPa at the rate of 0.02 MPa/min when the temperature reached 90 °C. After insulation was over, the composite laminates were then cooled down to 70 °C at 1.5 °C/min. The pressure relief was started after cooling down to 70 °C. The microwave curing process only occurred within the vacuum-bag pressure. The process temperature curve, except for air cooling period, is the same as the autoclave temperature curve.

Figure 2b shows the vibration pretreatment microwave curing process curve. The composite material was heated from room temperature to pretreatment temperature (80 °C, 90 °C, 100 °C) at 2 °C/min. After a certain vibration pretreatment time (10 min, 30 min, 50 min), a microwave was used to warm up to 130 °C and the dwell time was 120 min to complete the subsequent curing process. The vibration pretreatment process was accompanied by different vibration accelerations (5 g, 10 g, 15 g), and the values of these factors are listed in Table 1.

### 2.3. Mechanical Properties and Microscopic Characterization

In this study, void content and interlaminar shear strength were selected to evaluate the curing quality. Four 10 mm × 10 mm specimens were taken in the middle part of the laminates that had undergone the different curing processes along the 0-degree direction. After inlaying, polishing and ultrasonic cleaning, the laminate cross-section was photographed using an ultra-deep field 3D microscope; finally, the pictures were imported into Image pro plus 6.0 software. The percentage of pore area to the sample area was used as the void content in this process, i.e., the void content calculation method followed Equation (1):

(1) $$\gamma =\frac{S_{\gamma }}{S_{a}}\;\times \;100\%$$

where γ is the void content, S_γ_ is the void area and S_a_ is the specimen area.

**Figure 3:**
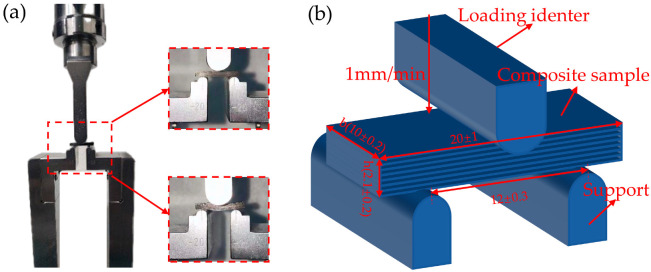
Short beam three-point bending test, (**a**) actual testing process, (**b**) sketch of short beam three-point bend.

At the same time, the interlaminar shear strength test was conducted according to national standard JC-T 773-2010 of the People’s Republic of China, and the test procedure and related parameters are shown in Figure 3.

Using the maximum load F_max_, the interlaminar shear strength τ was calculated from the following Equation (2):

(2) $$\tau =\frac{3}{4}\;\times \;\frac{F_{\max}}{\mathrm{bh}}$$

where b and h are specimen width and specimen thickness, respectively, and the units for both are mm.

## 3. Results and Discussion

### 3.1. Interlaminar Shear Strength Analysis

**Figure 4:**
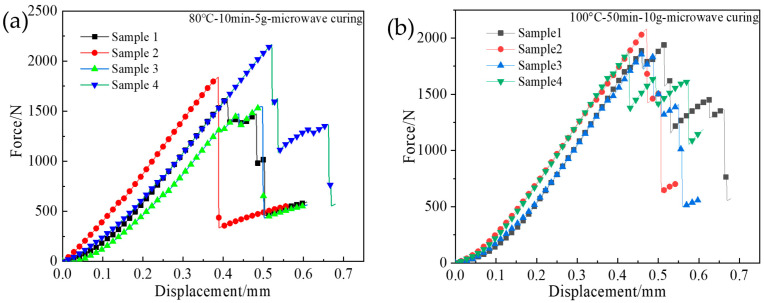
Three-point bending force–displacement curve of four specimens, (**a**) 80 °C-10 min-5 g-microwave curing process, (**b**) 100 °C-50 min-10 g-microwave curing process.

Figure 4 shows the three-point bending force–displacement curves of the four specimens during the vibration pretreatment parameters of 80 °C-10 min-5 g and 100 °C-50 min-10 g-microwave curing, which are the maximum and minimum values in nine sets of experiments, respectively. It can be seen that the force and displacement are approximately linear until the force reaches its maximum value, while after the force reaches its maximum value, the composite material loses its resistance. Therefore, the force value suddenly decreases. The average value of the interlaminar shear strength in the process is calculated using Equation (2). The experimental results of the interlaminar shear strength of the specimens under different vibration pretreatment microwave curing processes are listed in Table 2. It can be seen that the maximum interlaminar shear strength is group 9 and the minimum interlaminar shear strength is group 1. The difference between the maximum value and the minimum value is 16.46 MPa.

The above experimental results were transformed into the mean and extreme difference values of interlaminar shear strength under three factors and three levels according to the calculation method of extreme difference analysis, where a larger extreme difference value proves that the factor had a greater influence on the experimental results, and the final analysis results are shown in Table 3.

Table 3 reflects the mean and extreme difference results of the interlaminar shear strength of the composite specimens under three factors and three levels of pretreatment temperature, pretreatment time and vibration acceleration. It can be seen that the extreme difference of vibration acceleration is 7.82, which is the maximum extreme difference among the three factors, and the extreme difference values of pretreatment temperature and pretreatment time are 5.64 and 4.14 in turn, respectively, which are relatively small. Thus, it can be concluded that the factors affecting the magnitude of the shear strength of the laminate are: vibration acceleration > pretreatment temperature > pretreatment time, in order.

In order to illustrate the degree of significance of the effect of each factor on the interlaminar shear strength, the results of the above analysis need to be further analyzed using ANOVA, and the results of ANOVA are shown in Table 4.

The significance of the j factor on the evaluation index is obtained using the magnitude of the F value. It is assumed that the significance level to be checked is α, when F_j_ is greater than or equal to F_j_ (f_j_,f_e_) (f_j_, f_e_ is degree of freedom), meaning that the influence of the j factor is significant; otherwise, the influence is insignificant. In this study, the significance levels were chosen as α = 0.1, α = 0.05, α = 0.025, which correspond to the degree of significance level, and the highest is extremely significant while the lowest is extremely insignificant. The ANOVA data from the orthogonal test can be obtained with calculation and analysis, as shown in Table 4. From the ANOVA results, it can be seen that because F = 15.62 < F_0.05_ (2,2) = 19, it was judged that the pretreatment temperature had an insignificant effect on the interlaminar shear strength. The effect of pretreatment time F = 7.52 < F_0.10_ (2,2) = 9 on the interlaminar shear strength results was determined to be extremely insignificant, indicating that the length of pretreatment time has a small effect on interlaminar shear strength in the vibration pretreatment microwave curing process. Vibration acceleration corresponds to F = 34.51 > F_0.05_ (2,2) = 19, indicating the factor for the significant effect on the interlaminar shear strength. The main reason for the above results is that in the process of resin flow, where the pressure is generated using vibration acceleration to further compact the composite material, some of the smaller diameter bubbles are crushed; the larger diameter bubbles, because of the introduction of the vibration energy field, increase their rise rate and were extracted by the vacuum system, which improved the interlaminar shear strength of the composite laminate through abatement and inhibition of the pores during the molding process.

Through the above analysis, it can be found that vibration acceleration and pretreatment temperature result in the highest mean value of the interlaminar shear strength of the components at level II, so 10 g is selected for vibration acceleration and 90 °C for pretreatment temperature. The mean value of the interlaminar shear strength of the components at level III is the highest regarding holding time, but compared with level II, its interlaminar shear strength only increased by 1.46%, and combined with ANOVA, it can be seen that pretreatment time is insignificant. Meanwhile, considering the curing cost of the composite material and shortening the curing time, the pretreatment time at level II was chosen. In summary, the optimal vibration pretreatment process parameters selected were a pretreatment temperature of 90 °C, pretreatment time of 30 min and vibration acceleration of 10 g, as shown in Table 5, to find the interlaminar shear strength values of the composite laminate prepared using the microwave curing process, autoclave curing process and optimal vibration pretreatment microwave curing process.

Table 5 shows that optimal vibration pretreatment microwave curing increases the interlaminar shear strength by 34.6% compared to the microwave curing process, and its value reaches the level of the 0.6 MPa autoclave process.

### 3.2. Void Morphology Statistics

Porosity is one of the most common defects in the molding process of composite components, which is mainly caused by air and dust entrained during the lay-up process, volatile gases generated during the curing process of the composite components, and moisture trapped within and between the layers. The void content plays a decisive role in the performance and safety of composite components [30,31,32]; it is typically less than 1% for aerospace structural components [33,34].

**Figure 5:**
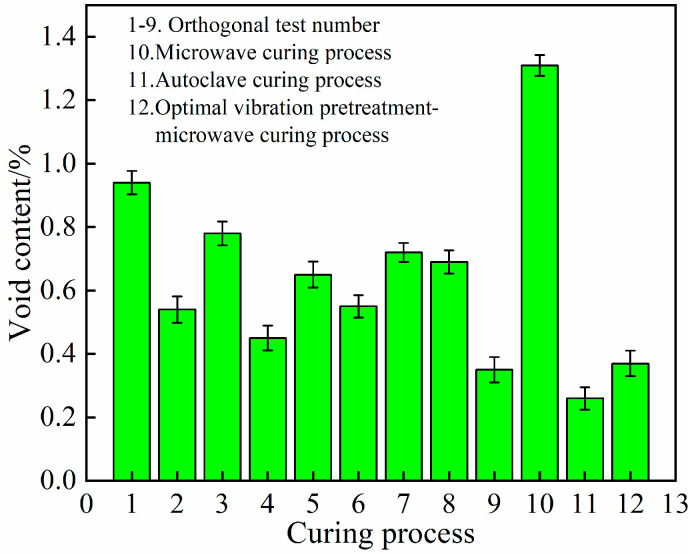
Void content of different curing processes.

The void content of the composite laminates prepared using the vibration pretreatment microwave curing process, the normal microwave curing process, the standard autoclave process and the optimized vibration pretreatment microwave curing process were statistically evaluated for each group in the orthogonal test table (Figure 5). It can be seen that the introduction of vibration pretreatment into the microwave curing process of the composite materials can significantly reduce the internal porosity of the components compared to the normal microwave curing process, and the optimized vibration pretreatment microwave curing process can reduce the porosity by up to 71.8% compared to the microwave curing process.

At the same time, the internal void content of the components can reach a level close to the 0.6 MPa autoclave process using specific process parameters, indicating that the introduction of vibration pretreatment has a significant effect on reducing and inhibiting the formation of defects during the microwave curing of aerospace composite components and controlling the void content to within 1%. Comparing the optimized vibration pretreatment microwave curing process with the standard autoclave process, the internal void content of the components was 0.37% and 0.26%, respectively. This proves that the optimization of the vibration pretreatment microwave curing process can achieve the same defect-suppression effect as the standard 0.6 MPa autoclave process. This result is consistent with the study of Guan [28,29].

**Figure 6:**
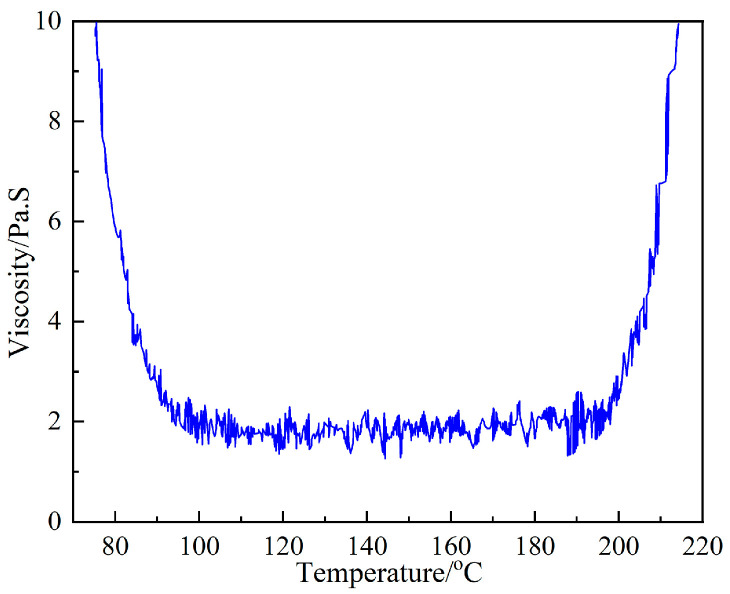
Viscosity–temperature curve of resin.

Figure 6 is the change curve of 602 resin viscosity with temperature. From the figure, it can be seen that the change trend of resin viscosity is first decreasing and then tends to stabilize and finally rise again. When the temperature exceeds 80 °C, the resin viscosity decreases rapidly, the decrease rate of viscosity gradually decreases and the resin gradually moves from a high viscosity state to a flow state. When the temperature rises to about 90 °C, the viscosity starts to reach its lowest point, and the viscosity value is about 2.1 Pas. When the temperature is within the range of 90–200 °C, the resin viscosity does not change much. After the temperature exceeds 200 °C, the viscosity of the resin increases rapidly and the rate of increase gradually accelerates, and the resin starts to change from the flow state to the glass state, at which time the viscosity of the resin rises rapidly again. The maximum curing temperature of the composite material used in this study is 130 °C. The reaction in the curing process is not only related to temperature but also positively correlated with time, and the higher the temperature, the more violent the curing reaction is within the same time range. Thus, 90 °C was chosen as the best time for pressurization without affecting the resin flow compaction and fiber infiltration process.

**Figure 7:**
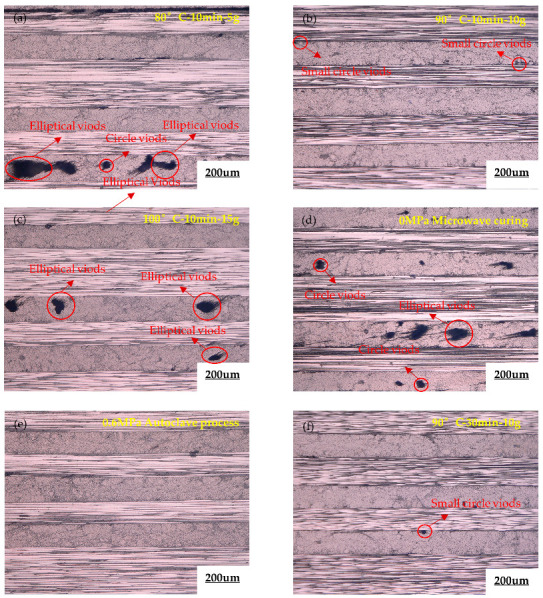
Microscopic morphology of vibration microwave curing process, microwave curing process, autoclave process, (**a**) 80 °C-10 min-5 g microwave curing process, (**b**) 90 °C-10 min-10 g microwave curing process, (**c**) 100 °C-10 min-15 g microwave curing process, (**d**) 0 MPa microwave curing, (**e**) 0.6 MPa autoclave process, (**f**) 90 °C-30 min-10 g microwave curing process.

Figure 7 shows the microscopic morphology of the interior of the component under different curing processes using an optical digital microscope (ODM). The scale bar is 200 um in the figure, and it can be seen that when the vibration pretreatment temperature and vibration acceleration are not selected properly, there are obvious large-sized voids inside the component, and the shape is mainly circular or elliptical. The reason for this phenomenon may be that the 602 epoxy resin does not reach the minimum viscosity at 80 °C, the resin mobility is poor and it cannot fully infiltrate with the carbon fiber bundle, as shown in Figure 7a.

As shown in Figure 7b, the overall pore shape under the curing process is a circle and the size is small, because the resin viscosity of the 602 resin at 90 °C is at its lowest point, and the resin and fiber wettability are very good. It only needs a short time to fully infiltrate the fiber, as shown in Figure 7c, where the microscopic shape under the curing process is circular or elliptical voids and the size is large. This phenomenon is mainly due to the fact that the viscosity of the composite material at 100 °C is past the minimum point and has increased to a certain extent, the resin is cured and cross-linked during the vibration process, the fiber infiltration is incomplete and some air bubbles are trapped in the pores and cannot be discharged. In terms of vibration acceleration, it can be seen from Figure 7a–c that the pore size has obvious differences under different vibration accelerations, and the equilibrium equation of action is shown in Equation (3).

(3) $$P_{v}{\;-\;P}_{r}{\;-\;P}_{e}{\;-\;P}_{\mathrm{vibr}}=\frac{{k\sigma }_{v}}{R_{v}}$$

P_v_ is internal pressure of the pore, P_r_ is hydrostatic pressure, P_e_ is external pressure (the vacuum-bagging pressure in the curing process is P_e_ = 0.1 MPa), P_vibr_ is pressure due to the vibration field, k is the surface tension coefficient, σ_v_ is pore surface tension and R_v_ is pore radius.

Under the condition of no vibration, the internal pressure of the pores inside the component is balanced with the external resin hydrostatic pressure, and the pores can exist stably and grow gradually with the increase in temperature. After the introduction of a vibration energy field, the equilibrium equation of the stable growth of pores is broken, and the energy excitation generated by the mechanical vibration makes the pore size change. The big pores decrease rapidly or even collapse in the action of the positive load generated by the vibration, and the composite component is further compacted. While the small pore expands and floats up after the direction of the vibration excitation is changed according to Stokes Equation (4), when the pore radius increases, it can quickly float up to the surface of the component in a shorter time and escape from the inside of the component under the action of a vacuum system:

(4) $$V_{b}=\frac{{2R}_{v}g∆\rho }{9\eta }$$

where V_b_ is the rise speed of the bubble, g is gravity acceleration, ∆ρ is the density difference between the resin and the gas inside the pore and η is resin viscosity.

As shown in Figure 7d, for the internal microscopic morphology of the components under microwave curing, it can be seen from the figure that the components contain big voids within and between the layers, and the number of pores is higher. The phenomenon is mainly due to the existence of only vacuum-bag pressure in the microwave curing process, and, due to insufficient pressure, the resin’s mobility and dense effect are poor. Figure 7e shows the microscopic morphology of the molded component under the 0.6 MPa autoclave process, and it can be seen that the high pressure of 0.6 MPa can fully drive the resin to infiltrate the fibers and gradually compact the laminate during the curing process. The components are molded with high quality, and the existence of pores can hardly be observed inside them. Figure 7f shows the microscopic morphology of the optimized vibration pretreatment microwave curing process, which is similar to the standard autoclave process. It is shown that the optimized composite molding process can achieve the same defect suppression as the 0.6 MPa autoclave process.

### 3.3. Fiber-Resin Bonding State

**Figure 8:**
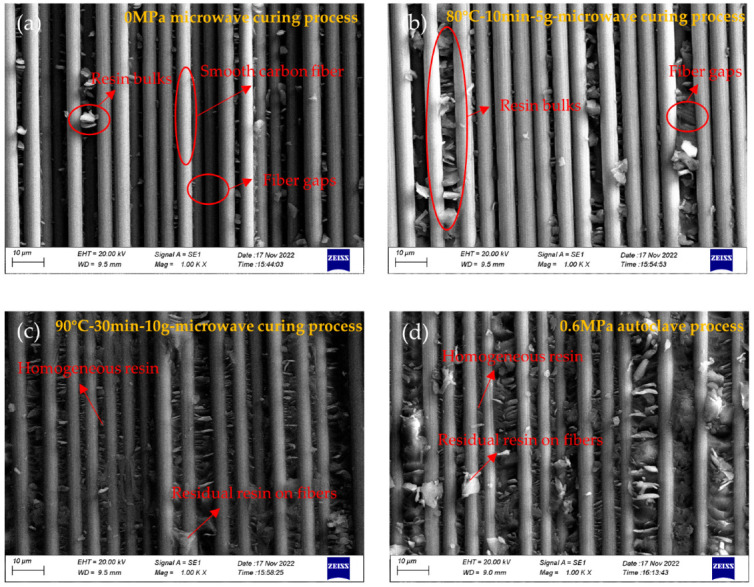
SEM of three-point bending fracture surface under different curing processes, (**a**) 0 MPa microwave curing process, (**b**) 80 °C-10 min-5 g microwave curing process, (**c**) 90 °C-30 min-10 g microwave curing process, (**d**) 0.6 MPa autoclave process.

To investigate the mechanism of the effect of a vibration energy field on the interlaminar shear strength, SEM was used to view the three-point bending section of the composite specimens under different curing process conditions, as shown in Figure 8. Figure 8a shows the section of the composite under microwave curing conditions, from which it can be seen that the fiber surface is smooth, the gap between the fibers is large, there is no resin filling and there is only a small number of resin bulks between the fibers, which indicates that microwave curing without vibration pretreatment has poor resin mobility and wettability, poor interfacial bonding properties, the existence of a large number of pores and even delamination defects, thus affecting the interlaminar shear strength. Figure 8b displays the pretreatment temperature of 80 °C, pretreatment time of 30 min and vibration acceleration of 5 g microwave curing, and from the figure it can be seen that, compared to Figure 8a, the gaps between the fibers are significantly smaller, and there are more resin bulks between the fiber gaps, which indicates that the vibration pretreatment conditions enhance the resin flow and compaction process in the microwave curing process. However, with the high viscosity of resin at 80°C and the effect of vibration acceleration, there is still room to improve the interlaminar shear strength. Figure 8c,d shows the SEM fracture surface of the specimens under the 90 °C-30 min-10 g microwave curing process and 0.6 MPa autoclave curing process. From the figure, it can be seen that their microscopic morphology is not significantly different, some residual resin exists on the fiber surface, the gaps in the carbon-fiber resin filling are relatively uniform, the void contents are significantly reduced and the resin impregnated well. This further explains why the specimens have similar shear strength between the optimal vibration pretreatment and microwave curing and 0.6 MPa autoclave curing processes and also shows that the vibration energy field can improve the resin impregnation in the carbon fiber and reinforcing fiber compaction process. Therefore, the resin and carbon fiber interface bonding state was improved, and the interlaminar shear strength of the specimens increased.

## 4. Conclusions

In this study, a three-factor, three-level combination of vibration pretreatment parameters, including pretreatment temperature, pretreatment time and vibration acceleration, was designed to adopt the orthogonal experimental method. This method was used to prepare resin-based composite laminates in different vibration pretreatment microwave curing processes. The influence of vibration pretreatment parameters on the performance of the microwave-cured molding of components was evaluated. Based on the results and discussion, the specific findings of this study are as follows.

Using interlaminar shear strength as the evaluation index of the orthogonal experiment, it is concluded from the extreme difference analysis that the influence order of interlaminar shear strength is: vibration acceleration > pretreatment temperature > pretreatment time. Combined with the manufacturing cost and manufacturing cycle of composite materials, it was determined that the optimal vibration pretreatment parameters are a pretreatment temperature of 90 °C, pretreatment time of 30 min and vibration acceleration of 10 g.

1. With the introduction of vibration pretreatment into the microwave curing process of T800/#602 aerospace composites, the composite laminate void contents were all below 1%, meeting the acceptance standards for aerospace structural components, further verifying the applicability of a vibration pretreatment microwave curing process for different material-forming systems.
2. The microscopic morphology of the specimens under different curing process conditions was characterized using ODM and SEM, and the reasons for the difference in interlaminar shear strength between the different curing processes were analyzed in terms of porosity and fiber-resin bonding state. The results show that the optimal vibration pretreatment microwave curing process reduced the void content by 71.8% and increased the interlaminar shear strength by 34.8% compared to the microwave curing process. The microscopic morphology and interlaminar shear strength levels were similar to those of the 0.6 MPa autoclave process (a standard process for aerospace components).

## Figures and Tables

**Table 1:** Orthogonal experimental design factors and levels L_9_ (3^4^) (1g = 9.8 m/s^2^).

Factor Levels	Pretreatment Temperature/°C	Pretreatment Time/min	Vibration Accelerate/g
Ⅰ	80	10	5
Ⅱ	90	30	10
Ⅲ	100	50	15

**Table 2:** Vibration pretreatment process orthogonal table and interlaminar shear strength results.

Test Number	Pretreatment Temperature (°C)	Pretreatment Time (min)	Vibration Accelerate (g)	Shear Strength (MPa)
1	80	10	5	67.18
2	80	30	10	78.51
3	80	50	15	71.83
4	90	10	10	80.87
5	90	30	15	75.21
6	90	50	5	78.37
7	100	10	15	73.37
8	100	30	5	74.01
9	100	50	10	83.64

**Table 3:** Mean and extreme difference results of interlaminar shear strength of laminates (Mpa).

		Factors	Pretreatment Temperature/°C	Pretreatment Time/min	Vibration Accelerate/g
	Means				
Levels					
Ⅰ			72.51	73.81	73.19
Ⅱ			78.15	75.91	81.01
Ⅲ			77.01	77.95	73.47
Extreme difference values			5.64	4.14	7.82

**Table 4:** Analysis of variance results for interlaminar shear strength of composites.

Factors	Sum of Deviation Squares	Degree of Freedom	Mean Square	F	Significance
Pretreatment temperature	53.404	2	26.702	15.62	Insignificant
Pretreatment time	25.712	2	12.856	7.52	Extremely insignificant
Vibration accelerate	118.034	2	59.017	34.51	Significant
Error	3.42	2	1.71		
F	F_0.1_(2,2) = 9		F_0.05_(2,2) = 19		F_0.025_(2,2) = 39

**Table 5:** Interlaminar shear strength values of composite laminates with different curing processes.

Curing Process	Interlaminar Shear Strength/MPa
Microwave curing	53.71
Optimal vibration pretreatment microwave curing	82.13
0.6MPa autoclave curing	84.12

## Data Availability

The data presented in this study are available upon request from the corresponding author.
